# Use of Electrical Penetration Graph Technology to Examine Transmission of ‘*Candidatus* Liberibacter solanacearum’ to Potato by Three Haplotypes of Potato Psyllid (*Bactericera cockerelli*; Hemiptera: Triozidae)

**DOI:** 10.1371/journal.pone.0138946

**Published:** 2015-09-25

**Authors:** Tariq Mustafa, David R. Horton, W. Rodney Cooper, Kylie D. Swisher, Richard S. Zack, Hanu R. Pappu, Joseph E. Munyaneza

**Affiliations:** 1 USDA-ARS, Yakima Agricultural Research Laboratory, Wapato, Washington, United States of America; 2 Department of Entomology, Washington State University, Pullman, Washington, United States of America; 3 Department of Plant pathology, Washington State University, Pullman, Washington, United States of America; University of Idaho, UNITED STATES

## Abstract

The potato psyllid, *Bactericera cockerelli* (Šulc) (Hemiptera: Triozidae), is a vector of the phloem-limited bacterium ‘*Candidatus* Liberibacter solanacearum’ (Lso), the putative causal agent of zebra chip disease of potato. Little is known about how potato psyllid transmits Lso to potato. We used electrical penetration graph (EPG) technology to compare stylet probing behaviors and efficiency of Lso transmission of three haplotypes of potato psyllid (Central, Western, Northwestern). All haplotypes exhibited the full suite of stylet behaviors identified in previous studies with this psyllid, including intercellular penetration and secretion of the stylet pathway, xylem ingestion, and phloem activities, the latter comprising salivation and ingestion. The three haplotypes exhibited similar frequency and duration of probing behaviors, with the exception of salivation into phloem, which was of higher duration by psyllids of the Western haplotype. We manipulated how long psyllids were allowed access to potato (“inoculation access period”, or IAP) to examine the relationship between phloem activities and Lso transmission. Between 25 and 30% of psyllids reached and salivated into phloem at an IAP of 1 hr, increasing to almost 80% of psyllids as IAP was increased to 24 h. Probability of Lso-transmission was lower across all IAP levels than probability of phloem salivation, indicating that a percentage of infected psyllids which salivated into the phloem failed to transmit Lso. Logistic regression showed that probability of transmission increased as a function of time spent salivating into the phloem; transmission occurred as quickly as 5 min following onset of salivation. A small percentage of infected psyllids showed extremely long salivation events but nonetheless failed to transmit Lso, for unknown reasons. Information from these studies increases our understanding of Lso transmission by potato psyllid, and demonstrates the value of EPG technology in exploring questions of vector efficiency.

## Introduction

Zebra chip disease of potato is thought to be caused by the phloem-limited bacterium ‘*Candidatus* Liberibacter solanacearum’ (Lso), transmitted by the potato psyllid, *Bactericera cockerelli* (Šulc) (Hemiptera: Triozidae) [1–4]. Zebra chip has caused millions of dollars in losses to the potato industry in the United States, Mexico, Central America, and New Zealand [3]. While zebra chip was first reported in Mexico in 1994 and in Texas in 2000 [3], it was not until 2011 that the disease reached the Pacific Northwest (ID, WA, OR), the major potato growing region of the US. [5–7].

Little is known about the mechanisms by which the potato psyllid transmits Lso to potato and other solanaceous species [3]. Plant diseases that are caused by insect-vectored pathogens generally are managed by controlling the insect vector, requiring knowledge about the dynamics of pathogen transmission and acquisition [8]. Success at preventing transmission of pathogens by controlling the insect vector will depend in part upon how rapidly the vector is able to transmit the pathogen following colonization of the host plant. Buchman et al. [8] showed that potato psyllid was capable of transmitting the zebra chip pathogen to potato within at least 6 h following colonization of the plant. However, Buchman et al. [8] did not monitor feeding behavior of the psyllids following insect colonization of the host plant, thus it is not known how much time was spent by psyllids actually feeding during the 6 h of access. Without information on actual time spent feeding, it is unclear what minimum length of time spent in feeding behaviors can lead to transmission.

The relationship between feeding behaviors and Lso transmission may be complicated further by the discovery of genetic variants, or haplotypes, of potato psyllid [9–10]. Three haplotypes occur regularly in potato fields of Idaho, Washington, and Oregon [10], where almost half of U.S. potato production is located. As we learn more about these haplotypes, it is becoming apparent that the genetic differences are associated with biological differences, including inter-haplotype differences in fitness [11–12], host use [13–16], and composition of their respective endosymbiont communities [17–18]. If Lso transmission efficiency, like other biological traits, proves to differ between psyllid haplotypes, then different psyllid management approaches to prevent transmission of Lso may be required. Therefore, knowledge of feeding behavior and Lso transmission efficiency of potato psyllid haplotypes is essential.

An innovative approach known as Electrical Penetration Graph (EPG) technology [19–20] is a useful tool to assess stylet probing behavior of plant-feeding Hemiptera, including psyllids. This technology allows observation and quantification of the feeding behavior of insects whose mouth parts are not visible while probing into plant tissues. EPG electrically records changes in resistance (in the form of consistent and repeated patterns referred to as “waveforms”) that relate to the different stylet penetration activities, providing information on stylet tip position in specific plant tissues and time spent at each location [21]. EPG has successfully been used to assess pathogen transmission by insect vectors [22–27], by correlating stylet penetration activities to acquisition and inoculation of pathogens [28–31].

This study used EPG technology to compare stylet probing behavior among psyllids of the Central, Western, and Northwestern haplotypes [9–10], and to examine the relationship between phloem-feeding activities and transmission of Lso to potato. Previous EPG studies with potato psyllid examined the behavior of individual haplotypes [32, 26–27]. Our first objective was to confirm that the three haplotypes occurring in the potato growing region of the Pacific Northwest exhibit the suite of probing behaviors and waveforms identified in earlier studies. We then statistically compared EPG results among haplotypes to look for haplotype-specific differences in probing behaviors that might translate into how efficiently a given haplotype transmits Lso.

Our second objective was to examine the relationship between stylet behaviors and probability of Lso transmission to potatoes. We determined whether probability of transmission is predicted by the amount of time a psyllid spends salivating into phloem tissue (thought to be associated with the E1 waveform; [27, 26]). This specific stylet behavior, among the various waveforms that have been identified for potato psyllid, has been suggested to play a key role in transmission of the phloem-limited Lso-bacterium [27, 26]. We manipulated the amount of time psyllids were allowed access to plants (“inoculation access period”, or IAP) to produce a range of E1 durations, which were then used to statistically describe the relationship between duration of E1 events and probability of transmission. These assays were also used to analyze whether transmission probability varied among haplotypes, to determine whether haplotypes differ in how efficiently they transmit Lso at a common length of time in phloem. Finally, the EPG data were used to identify the minimum amount of time salivating into phloem that leads to transmission. These data are useful in quantitatively showing how rapidly an infected psyllid is capable of transmitting Lso to potato once phloem tissues have been accessed.

## Materials and Methods

### Sources of insects

Lso-free and Lso-infected potato psyllid colonies of three haplotypes were established at the USDA-ARS facility in Wapato, WA (46°26′44″N, 120°25′19″W), with insects collected from Weslaco, Texas (26°9′33″N, 97°59′15″W) (Central haplotype), Albany, California (37°53′13″N, 122°17′52″W) (Western haplotype), and Prosser, Washington (46°12′25″N, 119°45′56″W) (Northwestern haplotype). No collection or import permit was required by Texas, California, or Washington; potato psyllid is not an endangered or protected species. The colonies were started with isofemale lines and were maintained on Atlantic potatoes under laboratory conditions of 25±1°C, 40±5% RH, and a photoperiod of 16:8 (L: D) h. Haplotype of psyllids was confirmed using high resolution melting analysis [9,16].

### Sources of plants

Certified disease-free potatoes (var. Atlantic) grown from seed tubers obtained from CSS Farms Inc. (Colorado City, CO) were used for this study. This variety was selected due to its high susceptibility to zebra chip [1, 33–34]. Tubers were planted in 0.5-L pots (Kord Products, Toronto, Ontario, Canada) filled with a soil medium consisting of 86% sand, 13.4% peat moss, 0.5% Apex time release fertilizer (J. R. Simplot Co., Lathrop, CA), and 0.1% Micromax micronutrients (Scotts Co., Marysville, OH), and grown in a greenhouse located at the USDA-ARS facility.

### Electrical penetration graph (EPG) recordings

EPG system wiring and recordings were conducted as described by Pearson et al. [27]. Psyllids that were to be monitored were first placed on ice to reduce mobility. The immobilized psyllids were then secured by gently gripping their fore- and hind wings with a pair of soft grip forceps (Bio Quip, Rancho Dominguez, CA) to wire the insects to the EPG system. An electrode consisting of a 3-cm long copper wire soldered to the head of a 3 mm diameter brass nail was used to wire the insects. One end of the electrode was attached to the psyllid pronotum using a hand-mixed silver glue adhesive. The silver glue adhesive mixture consisted of 1:1:1 (v: v: w) of water-based white household glue, water and silver flake (Inframat Advanced Materials LLC, Manchester, CT, USA). The other end of the electrode consisted of a 3-cm long and 25.4-μm diameter gold wire (sold as a 0.0010 in.; Sigmund Cohn., Mt. Vernon, NY, USA) attached to the copper wire portion of the electrode. The wired insects were left at room temperature for a 30-min recovery and wiring adjustment period on a small styrofoam pinning board and without plant material. After the recovery period, the electrode was inserted into the EPG amplifier and the tethered psyllid was placed on the abaxial surface of a leaf of a potted potato plant in a Faraday cage, and stylet probing behavior was recorded. The EPG recordings were obtained under controlled conditions of 25±1°C, 40±5% RH, and a photoperiod of 16:8 (L: D) h. Waveforms were acquired using a four-channel AC-DC EPG monitor (Backus and Bennett [20]); EPG Equipment Co., Otterville, MO); thus, recordings could be done simultanousily for a maximum of four psyllids. The EPG data were digitized using a WinDaq DI-720 analog-to-digital (A-D) board and recorded with WinDaq Pro+ acquisition software (DATAQ Instruments, Akron, OH) at a sample rate of 100 Hz, input impedances (Ri) of 10^9^ Ohms (Ω), and DC substrate voltage.

### Validation of EPG methods and comparison of haplotypes

We first verified that our methods produced waveform results for all three haplotypes similar to those obtained by Pearson et al. [27] for psyllids of the Central haplotype. Data from these assays were also used to statistically compare probing activities among haplotypes. Individual psyllids of each haplotype were collected randomly from the laboratory colonies and starved for 2–3 h at 4°C. Following starvation, the psyllids were tethered to electrodes as described above and placed on the abaxial surface of leaves of potato plants. Probing behavior for each psyllid was recorded for 24 h. Sample size was 16 insects per haplotype.

Pearson et al. [27] reported six waveform families produced by potato psyllid feeding on potato. These waveform families were consistent in appearance with those produced by potato psyllid in other studies [32, 26] and included: family A, initial penetration and saliva secretion; family B, penetration of epidermal cells; family C, secretion of most of the salivary sheath and stylet pathway in mesophyll/parenchyma; family D, initial contact with phloem cells; family E, salivation and ingestion activities in phloem cells, with two types, E1, salivation (26, 27), and E2, phloem sap ingestion (26, 27); and family G, xylem ingestion. In the present study, the total numbers of waveform events and time spent in each of these waveforms were recorded and compared statistically among haplotypes. The amount of time to first intercellular stylet probe (C), xylem sap ingestion (G), phloem salivation (E1), and phloem ingestion (E2) were each also estimated from waveforms and compared among haplotypes.

### Assessing Lso transmission efficiency among haplotypes

**Fig 1:**
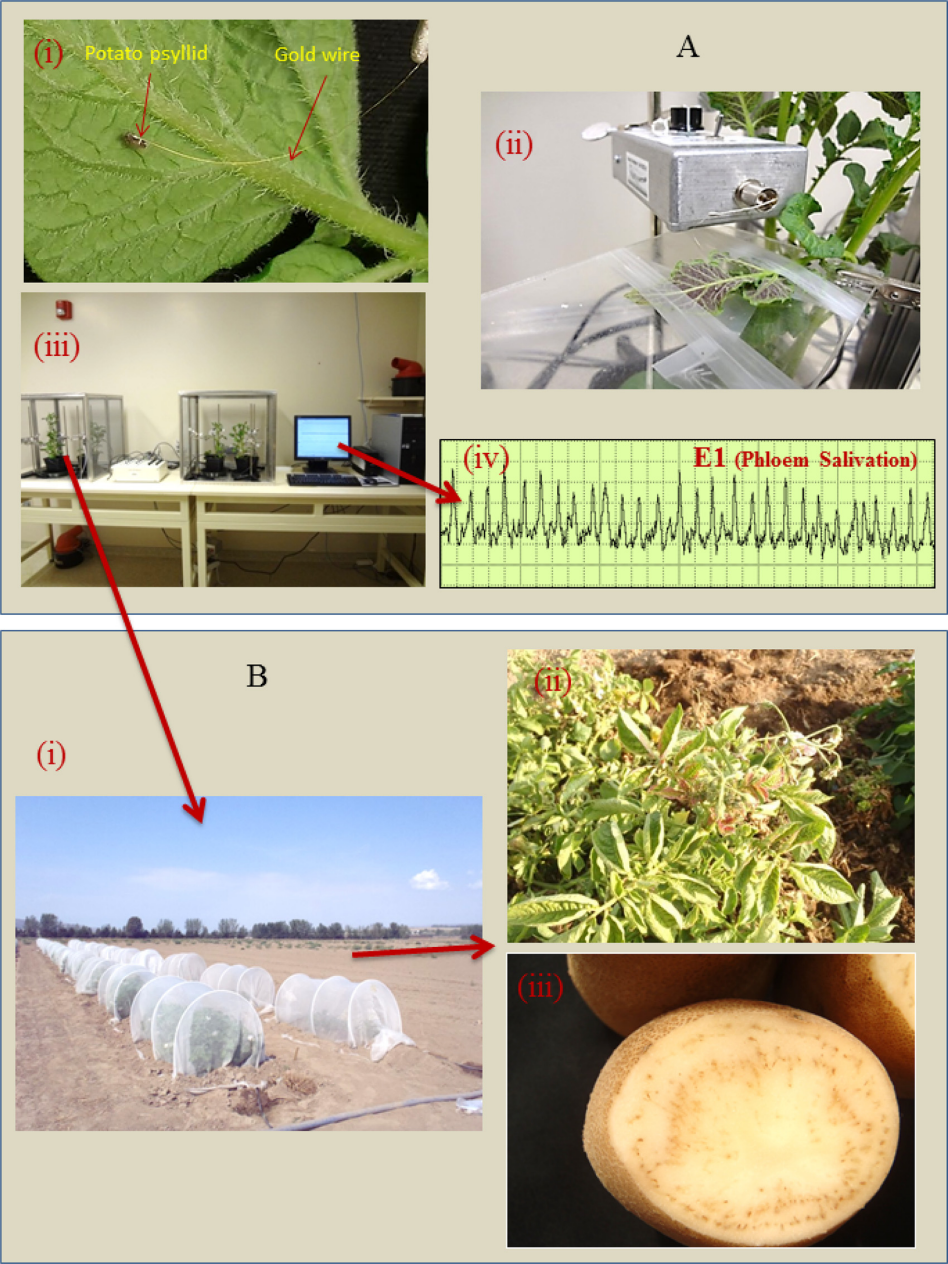
Steps used in examining ‘*Candidatus* Liberibacter solanacearum’ transmission as a function of phloem activities. (A) Electrical penetration graph (EPG) Assays: (i) A potato psyllid tethered to a gold wire on a potato leaf, the wire is attached on an insect electrode, (ii) An insect electrode inserted into the amplifier, (iii) An EPG recording station consisting of two Faraday cages with potato plants, an AC-DC monitor, and a computer, (iv) Quantifying the duration of E1 activities exhibited by infected psyllids as illustrated by a typical phloem salivation waveform. (B) Field-monitoring of potato plants from the EPG assays to determine whether plants develop zebra chip symptoms: (i) Field cages, (ii) Zebra chip foliar symptoms consisting of leaf curling and discoloration, (iii) Characteristic zebra chip symptoms in potato tuber.

Once we had validated our EPG methods, we then examined the relationship between duration of stylet behaviors and transmission of Lso to potato. We compared the EPG waveforms of psyllids that successfully transmitted Lso and waveforms of infected psyllids that had failed to transmit Lso. We limited our analyses to an examination of how variation in the E1 waveform affected probability of transmission, as both Pearson et al. [27] and Sandanayaka et al. [26] suggested that it is this waveform (thought to depict salivation into the phloem) that plays the key role in inoculation. Completion of this objective required a series of steps (Fig 1) which we describe in the following paragraphs, culminating in a field-examination of psyllid-challenged plants to determine presence of zebra chip disease and Lso.

Psyllids of each of the three haplotypes were collected from Lso-infected colonies and starved for 6 h at 4°C prior to EPG hook-up (Fig 1Ai). Each tethered psyllid was placed on the abaxial surface of a potato leaf in a Faraday cage, and given an inoculation access period of 1, 2, 3, 4, 5, 6, 12, or 24 h, during which stylet probing behavior was recorded with EPG (Fig 1Aii). By manipulating IAP in this manner, we were able to produce a diverse series of waveform patterns (notably in frequency and duration of the E1 waveform). At the end of each assay the psyllid was tested for presence of Lso by PCR (methods described below) to ensure that the subsequent statistical analyses included only data from infected insects.

Each plant from the EPG portion of the assays was then monitored for development of zebra chip symptoms. Because symptoms associated with zebra chip develop more readily in field-grown plants than in potted plants, all plants were transplanted into small field cages located out-of-doors (Fig 1Bi). Transplants were monitored for development of zebra chip symptoms over the course of a 6–8 week period [8, 35]. A subsample of symptomatic plants (Fig 1Bii and 1Biii) was tested with PCR as described by Munyaneza et al. [36] to confirm that plants having symptoms were indeed infected with Lso. In addition, all asymptomatic plants were tested with PCR in the event that infection had not yet produced visual symptoms. Thus, for each psyllid that was assayed, the following data were obtained: (1) psyllid haplotype, (2) EPG recording results (Fig 1A), (3) confirmation that the psyllid was carrying the Lso-pathogen, and (4) whether the insect had successfully transmitted the Lso pathogen to the target plant (Fig 1B). Taken together, these results allowed us to quantify the association between duration of phloem-related feeding activities, haplotype differences in these activities, and presence of disease in plants. Twenty psyllids were used for each haplotype and each inoculation access period, for a total of 480 insects.

The presence or absence of Lso in psyllid and plant samples was determined using PCR to amplify a region of 16S rDNA. DNA from psyllids was extracted using the cetyltrimethlyammonium bromide (CTAB) method of Zhang et al. [37], whereas DNA extraction from plant samples used the CTAB method of Pastrik and Maiss [38] with a slight modification as described by Munyaneza et al. [36]. PCR was performed using the primer pair OA2/OI2c [39, 2] with Go Taq reagents (Promega, Madison, WI) under the thermal cycling conditions described by Munyaneza et al. [36]. PCR products were separated on 1.5% agarose gels containing ethidium bromide for visualization. The presence of Lso was indicated by presence of the expected 1168-bp amplicon.

### Data Analysis

The average duration and number of events of intercellular stylet penetration/pathway phase (C); xylem ingestion (G); initial contact with phloem tissue (D), salivation into phloem sieve elements (E1), and phloem sap ingestion (E2) probing events were compared among psyllid haplotypes using the GLIMMIX procedure of SAS 9.3 [40]. Data were examined for heterogeneity of variance and for non-normality of errors by inspecting residual and normal quantile-quantile plots, respectively. Time to first intercellular stylet probe, and time to first xylem ingestion (G), phloem salivation (E1) and phloem ingestion (E2) following the first intercellular stylet probe were also compared among haplotypes using GLIMMIX. For these analyses, we specified an underlying gamma distribution using the DIST = GAMMA option; this distribution often is used to model time to occurrence of an event [41].

We examined the interactive effects of haplotype and IAP on probability of plant infection and on probability that the psyllid had accessed phloem tissues as a 3 x 8 (haplotype x IAP) factorial model in GLIMMIX, specifying the DIST = BINARY option. Lastly, we used logistic regression to examine the relationship between duration of E1 (phloem salivation) events and probability of successful transmission of Lso. The analyses were done using PROC LOGISTIC [42]. Haplotype was included as a class variable to statistically examine whether haplotypes were similar in efficiency of transmission for a given E1 duration.

## Results

### Validation of EPG methods and comparison of haplotypes

**Fig 2:**
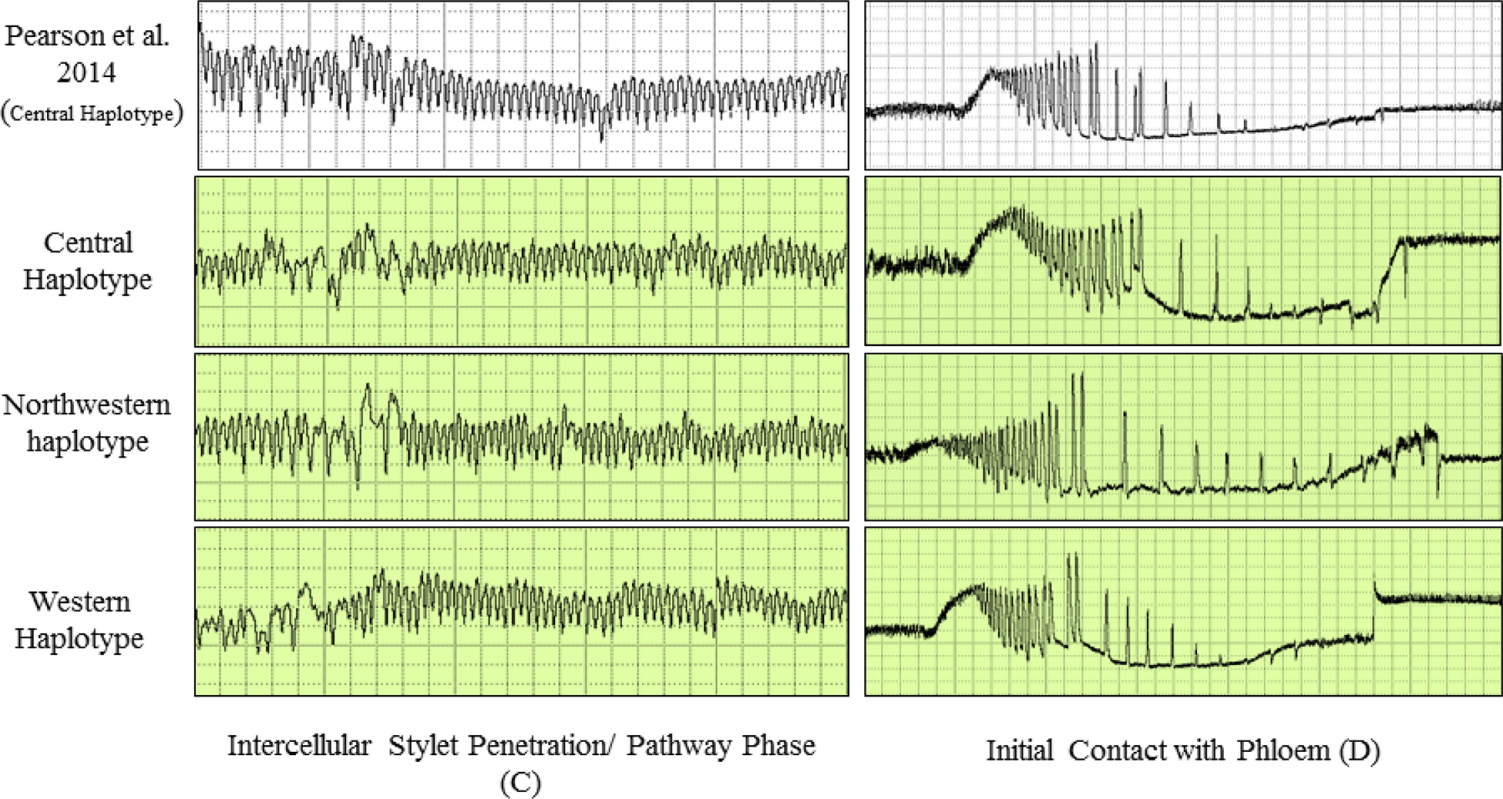
Electrical penetration graph (EPG) waveforms produced by potato psyllids of three haplotypes during intercellular stylet penetration/pathway phase (C) and initial contact with phloem tissue (D). The waveforms are compared to characteristic EPG waveforms identified and described by Pearson et al. [27] for psyllids of the Central haplotype.

**Fig 3:**
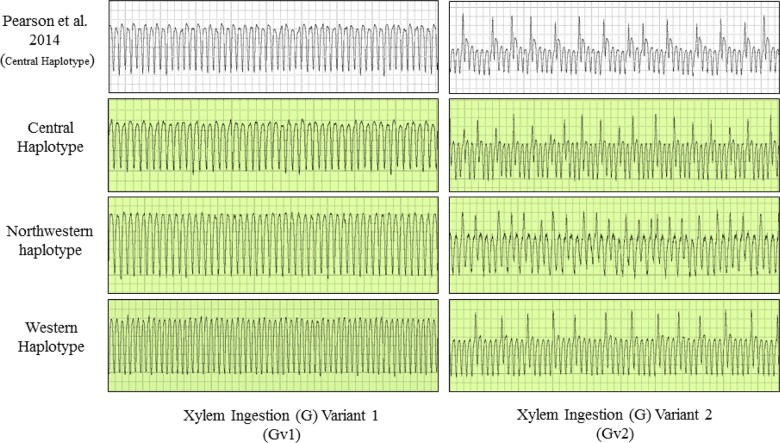
Electrical penetration graph (EPG) waveforms produced by potato psyllids of three haplotypes during the ingestion of xylem sap (G), with variants (Gv1 and Gv2). The waveforms are compared to characteristic EPG waveforms identified and described by Pearson et al. [27] for psyllids of the Central haplotype.

**Fig 4:**
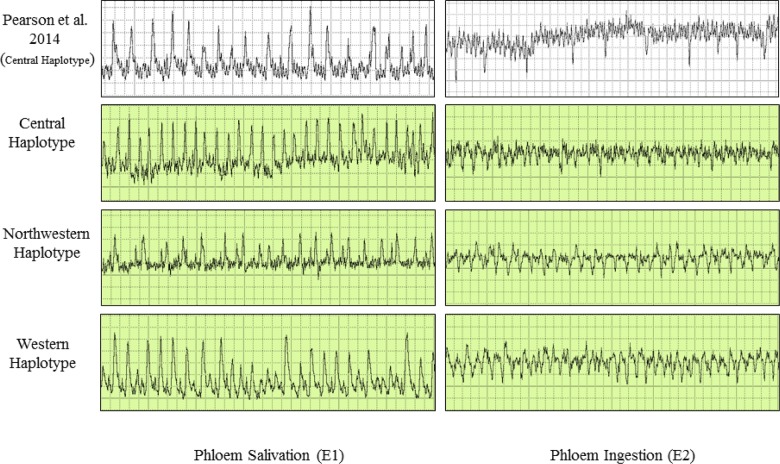
Electrical penetration graph (EPG) waveforms produced by potato psyllids of three haplotypes during salivation into phloem sieve elements (E1), and ingestion of phloem sap (E2). The waveforms are compared to characteristic EPG waveforms identified and described by Pearson et al. [27] for psyllids of the Central haplotype.

Waveforms produced by infected psyllids of all three haplotypes were similar in appearance to those shown by Pearson et al. [27] for uninfected psyllids of the Central haplotype (Figs 2–4). From that earlier work, which correlated waveform patterns with actual location of stylets in plant tissues [27], we identified in our study representative examples of all stylet activities summarized in the earlier publication. Those waveforms and the feeding behaviors each is assumed to be correlated with comprised the following (Figs 2–4): (C) intercellular style penetration/pathway phase, (D) initial contact with phloem tissue, (G) ingestion of xylem sap, (E1) salivation into phloem sieve elements, and (E2) ingestion of phloem sap.

**Fig 5:**
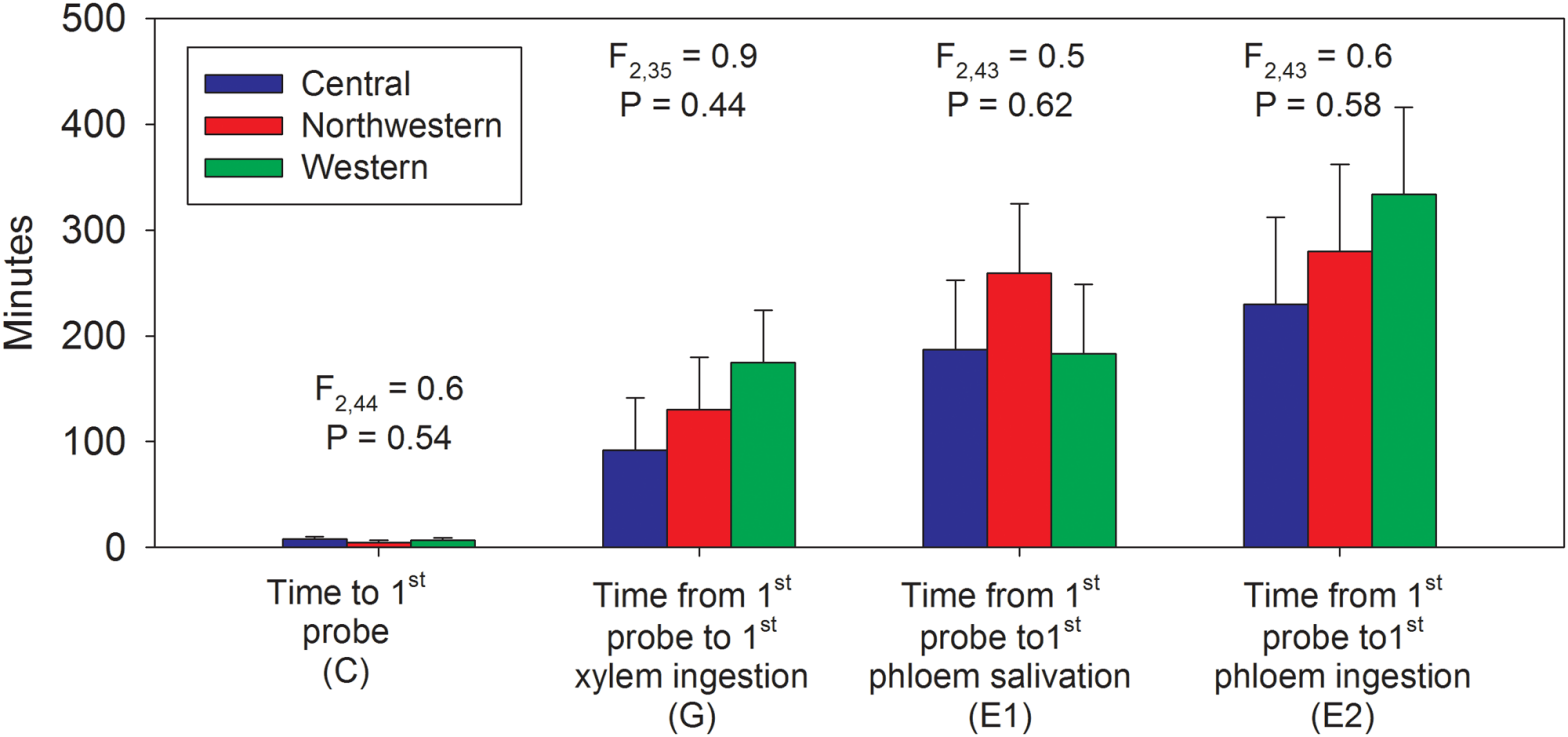
Mean (SEM) number of minutes required by psyllids of each haplotype to begin probing, and to initiate ingestion and salivation activities following onset of probing. F- and P-statistics summarize haplotype effects for each feeding behavior.

**Fig 6:**
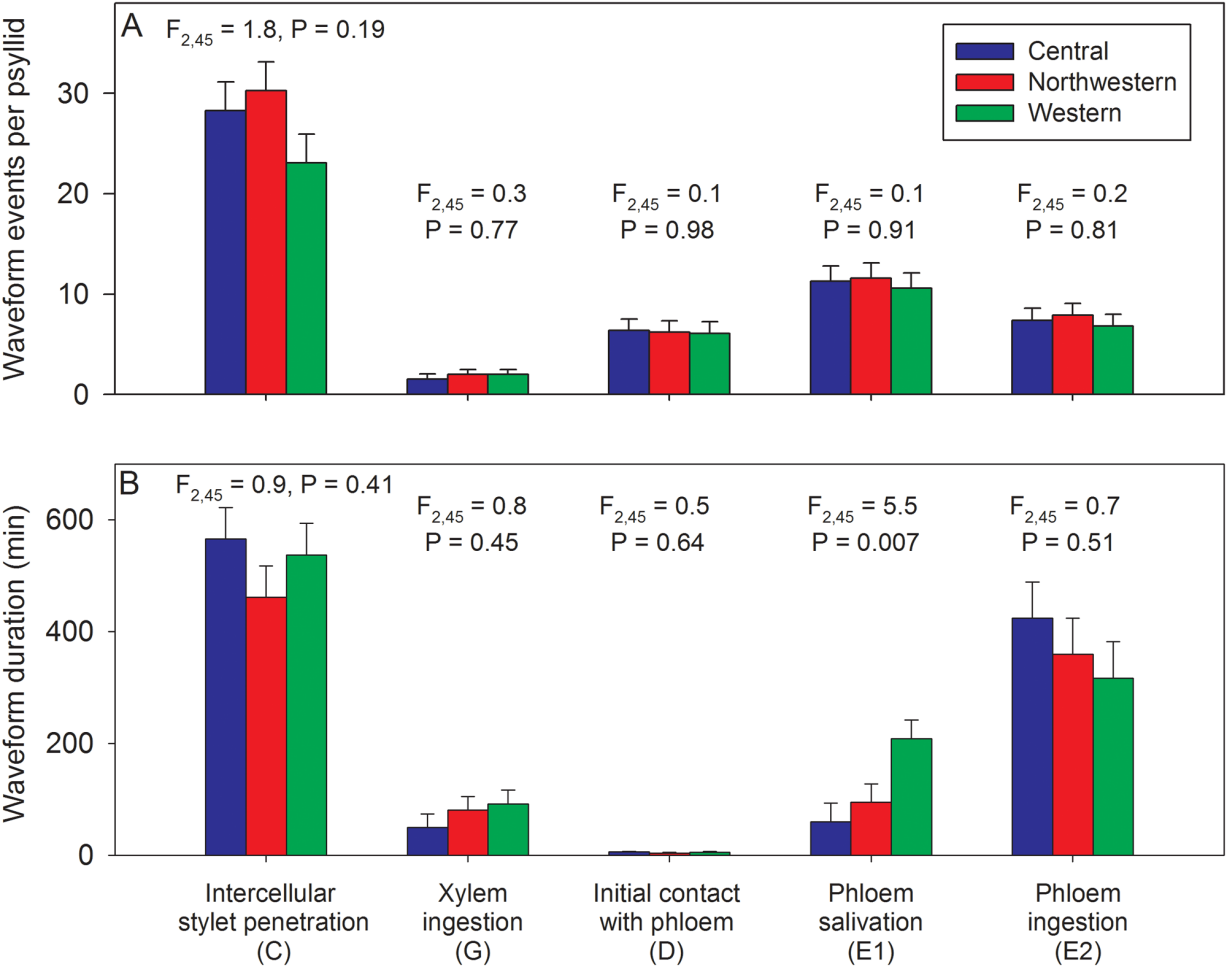
Mean (SEM) number of different stylet probing events per psyllid (A) and mean (SEM) number of minutes spent in each behavior (B) over the duration of a 24 h assay for psyllids of each haplotype. F- and P-statistics summarize haplotype effects for each feeding behavior.

The EPG waveforms showed that the first probe was initiated on average within a few minutes (< 10 min) following onset of the assay (Fig 5). The mean number of minutes between onset of probing and onset of xylem ingestion (90–170 min), onset of phloem salivation (180–260 min), and onset of phloem ingestion (230–335 min) was not affected by haplotype (Fig 5; F- and P-statistics for haplotype effects are provided above each trio of bars). Psyllids of each haplotype often exhibited multiple occurrences of each waveform type within an individual (24 h) assay, particularly for intercellular stylet penetration (C) which averaged 23–30 separate occurrences per psyllid (Fig 6A). Individual psyllids averaged approximately 10 separate E1 events over the course of the 24 h assay, irrespective of haplotype (Fig 6A). There were no statistical differences among haplotypes in number of occurrences of a given stylet behavior over the duration of a 24 h assay (Fig 6A; F- and P-statistics provided above each trio of bars). The mean duration per psyllid of each behavior was estimated after first summing durations over multiple occurrences for those psyllids which exhibited multiple occurrences of a given behavior. Results showed that the largest amount of time was spent in intercellular stylet penetration (C) and phloem ingestion (E2), irrespective of haplotype (Fig 6B). The only haplotype effect noted in this assay was for phloem salivation activities (E1), with results showing that psyllids of the Western haplotype exhibited a modestly longer average duration of this behavior than psyllids of the other haplotypes (Fig 6B). This tendency for the Western haplotype to have E1 waveforms of somewhat longer duration than noted for the other haplotypes was also found in the Lso-transmission assay (summarized in the following section).

### Assessing Lso transmission efficiency among haplotypes

**Fig 7:**
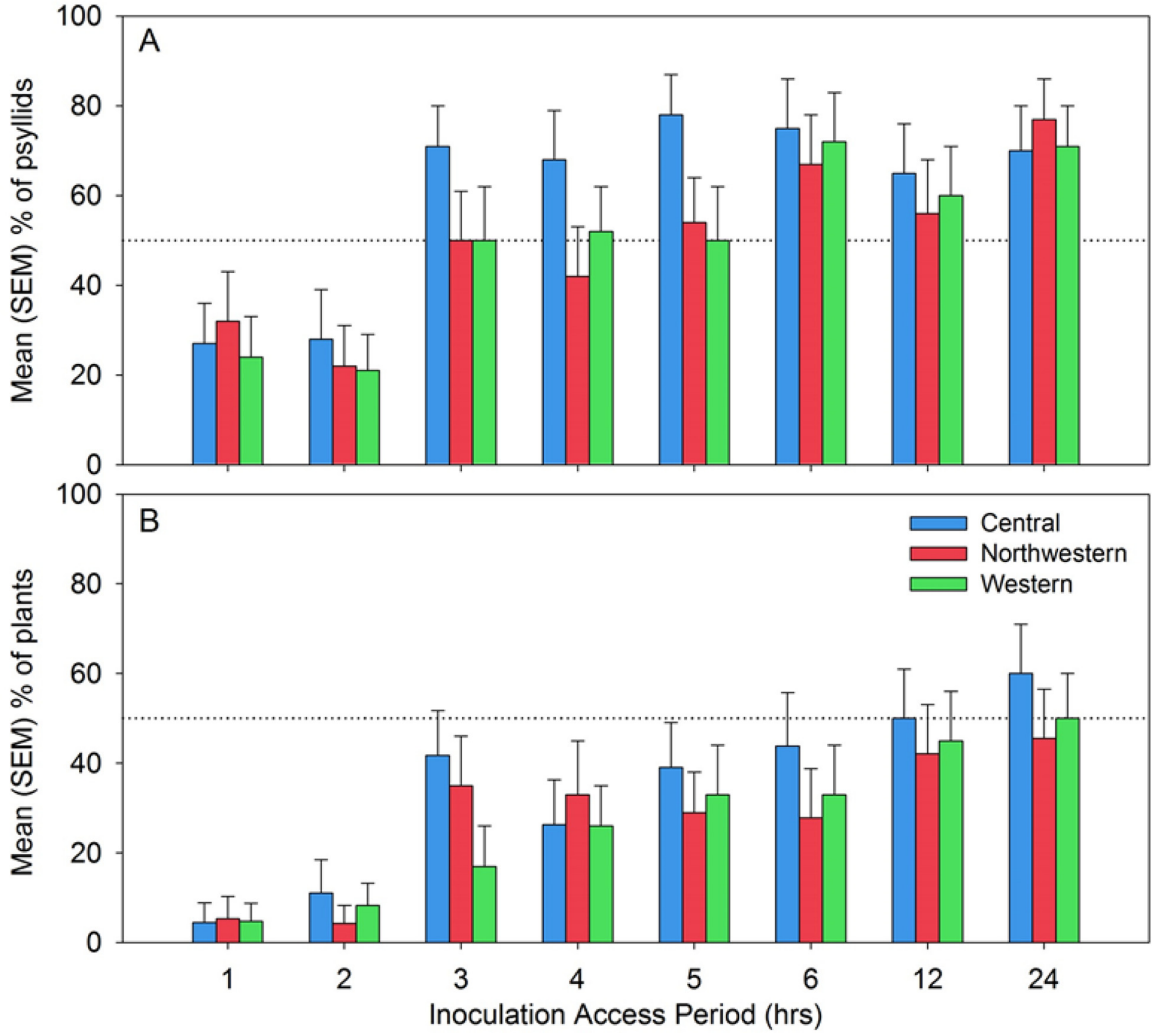
Mean (SEM) percentage of psyllids of each haplotype which fed in phloem tissue as a function of inoculation access period (A); and, mean (SEM) percentage of psyllids of each haplotype which successfully transmitted Lso to potato as a function of inoculation access period (B).

Probability that a psyllid reached phloem tissues during its probing activities varied between approximately 0.25–0.30 at the lowest IAP of 1 h to over 0.70 at the highest IAP of 24 h (Fig 7A). Mean probability was significantly affected by IAP (Fig 7A; F_7, 467_ = 7.7, P < 0.0001) but not by haplotype (F_2, 467_ = 2.3, P = 0.10) or the haplotype x IAP interaction (F_14, 467_ = 0.4, P = 0.97). Profile contrasts were extracted to compare mean probabilities between adjacent levels of the IAP factor, and showed that there was a significant increase between the 2 h and 3 h levels of IAP in probability that a psyllid had fed in phloem tissues (Fig 7A; F_1, 467_ = 14.1, P = 0.0002). No other contrasts were significant, indicating that probabilities were statistically flat between 1 h and 2 h IAP, and between 3 h and 24 h IAP (Fig 7A).

Probability of plant infection was consistently lower across all IAP levels than probability that the psyllid had fed in phloem tissues, indicating that a percentage of infected psyllids which reached phloem tissues nonetheless failed to induce disease in the potato plant (Fig 7B; contrast the results in Fig 7B with the probabilities in Fig 7A). Mean probability of plant infection increased with IAP (Fig 7B; F_7, 464_ = 6.2, P < 0.0001), again with a statistically significant increase in probabilities only between the 2 and 3 h IAP levels (Fig 7B; F_1, 464_ = 8.5, P = 0.004). Haplotype effects were non-significant (F_2, 464_ = 0.9, P = 0.41), as was the haplotype x IAP interaction (F_14, 464_ = 0.3, P = 0.99).

**Fig 8:**
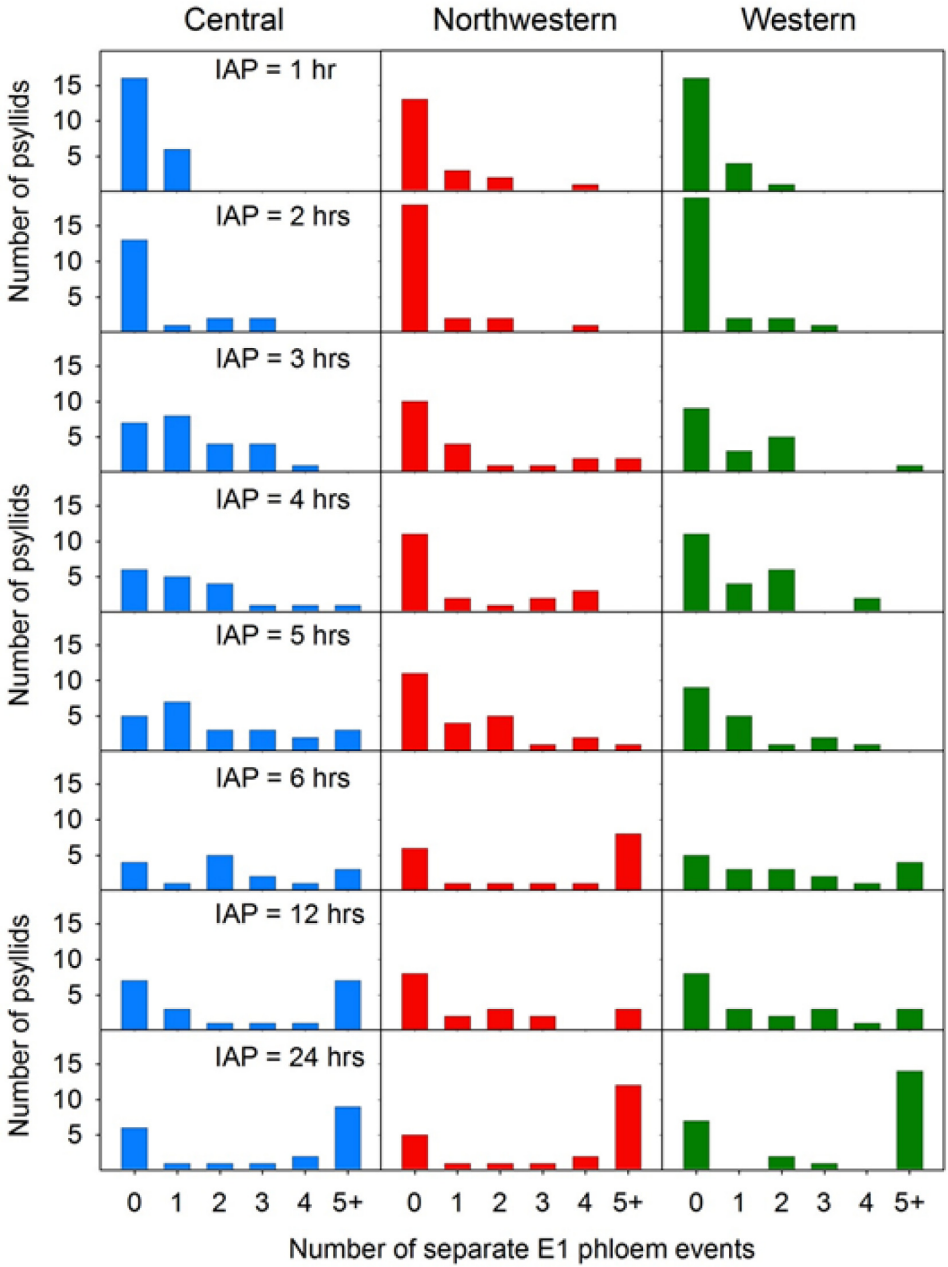
Histograms showing frequency of psyllids of each haplotype which exhibited 0, 1, 2, 3, 4, or 5+ separate E1 events over the course of an assay as a function of length of the assay (inoculation access period, or IAP).

**Fig 9:**
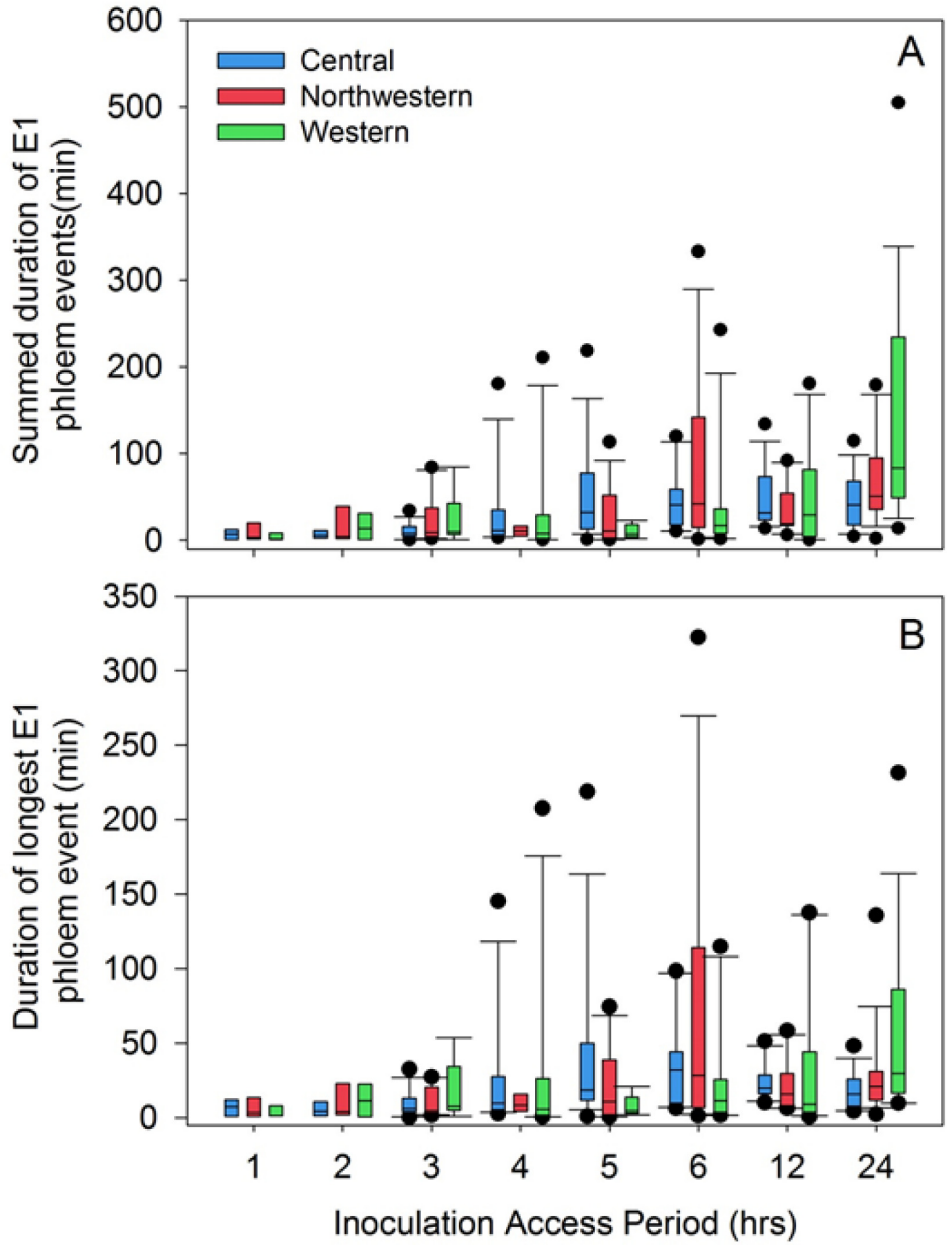
Box plots showing duration of E1 events for three haplotypes of potato psyllid as a function of inoculation access period. (A) Durations were summed across multiple events for each psyllid; (B) longest duration E1 event for each psyllid. Figures exclude psyllids failing to reach the phloem. Box plots show boundaries of 25^th^ and 75^th^ percentiles (box boundaries), median (line within each box), 10^th^ and 90^th^ percentiles (whiskers), and outlying points (circles).

The observation that probability of Lso-transmission (Fig 7B) was consistently lower than probability that the psyllid had fed in phloem tissues (Fig 7A) prompted us to look for patterns in phloem salivation (E1 waveform) that may have affected transmission. By varying IAP between 1 and 24 h, we were able to affect frequency with which psyllids exhibited the E1 waveform (Fig 8) as well as duration of those E1 events, with the latter expressed either as total duration summed over multiple E1 occurrences per psyllid (Fig 9A) or as the longest E1 event for each psyllid (Fig 9B). Not surprisingly, the proportion of psyllids exhibiting more than a single E1 event during an assay increased with increasing IAP, to the extent that a large proportion of psyllids of all three haplotypes at the 24 h IAP exhibited more than 5 separate E1 events over the course of the 24 h (Fig 8; bottom 3 panels). Long-duration E1 events became increasingly common with increasing IAP (Fig 9). Several psyllids exhibited bouts of continuous E1 behavior exceeding 200 min at the higher values of IAP (Fig 9B).

**Fig 10:**
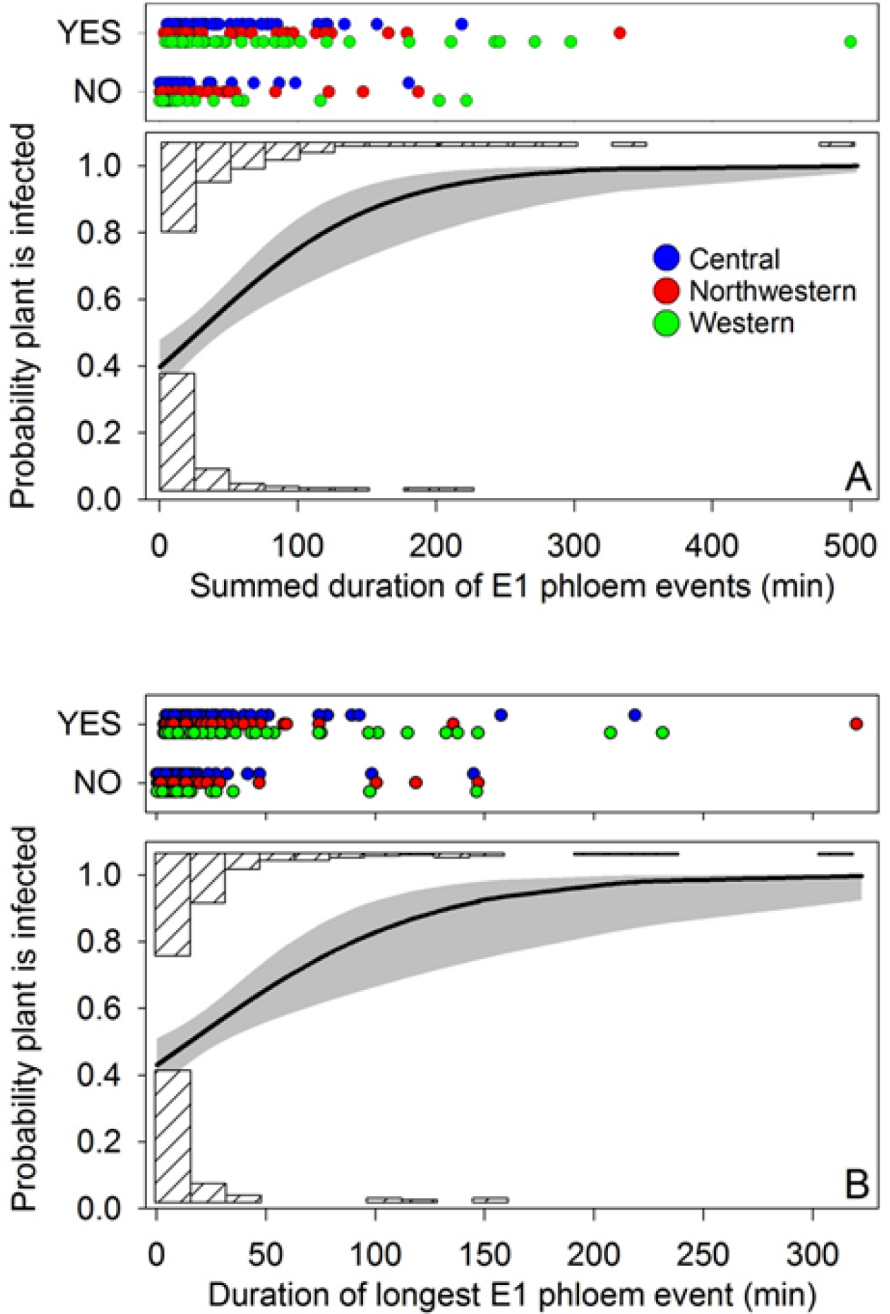
Results of logistic analysis showing relationship between probability of Lso-transmission and duration of E1 events, expressed either as the total duration of E1 events for each psyllid (A) or as duration of the longest E1 event for the psyllid (B). Analyses exclude psyllids which failed to reach phloem tissues. Solid lines indicate fitted regression lines (pooling haplotypes) with 95% confidence limits around the regression shown by gray shading. Colored circles in scatter plot show E1 durations that led to plant infection (“YES”) or failed to lead to plant infection (“NO”) for the three haplotypes; each circle represents an individual psyllid. Downward facing, cross-hatched histograms show number of psyllids (pooled haplotypes) producing E1 durations that caused plant infection, while upward facing histograms show number of psyllids producing E1 durations which failed to cause plant infection.

Probability of plant infection was modeled as a function of E1 duration using logistic regression (Fig 10; data used in the logistic analyses shown in S1 Table). The analyses included all psyllids exhibiting at least one E1 event (thus excluding psyllids which failed to reach the phloem) irrespective of IAP. We examined total duration of E1 events for those psyllids which showed multiple E1 occurrences within an assay (Fig 10A), and, for each psyllid, its longest E1 duration (Fig 10B). Haplotype and the haplotype × duration interaction were not significant in the logistic models (total E1 duration: haplotype, χ^2^ = 0.9, df = 2, P = 0.65; haplotype × duration, χ^2^ = 2.4, P = 0.31; longest E1 duration: haplotype, χ^2^ = 0.6, df = 2, P = 0.75; haplotype × duration, χ^2^ = 1.2, P = 0.55), so the final logistic models were fitted without either effect (Fig 10A and 10B). Probability of infection for the pooled haplotype sample is shown by the solid black line in each panel, with the gray shading indicating the 95% confidence limits. In both analyses, the duration effect was highly significant (Fig 10A: χ^2^ = 17.7, df = 1, P < 0.0001; Fig 10B: χ^2^ = 12.1, df = 1, P < 0.0001), and showed that probability of plant infection increased with increasing duration of E1 events. Transmission of Lso occurred in some instances even following a very short interval of salivation, shown by the large numbers of psyllids at short durations which nonetheless induced plant disease (the “YES” psyllids in the scatter plots of both panels). However, there were also a number of infected psyllids which failed to produce disease in plants despite extremely long intervals of E1 behavior, as shown by the occurrence of long-duration psyllids labeled “NO” in the scatter plots (Fig 10). We discuss possible explanations for the occasional absence of successful transmission associated with long-duration E1 events in the Discussion.

**Fig 11:**
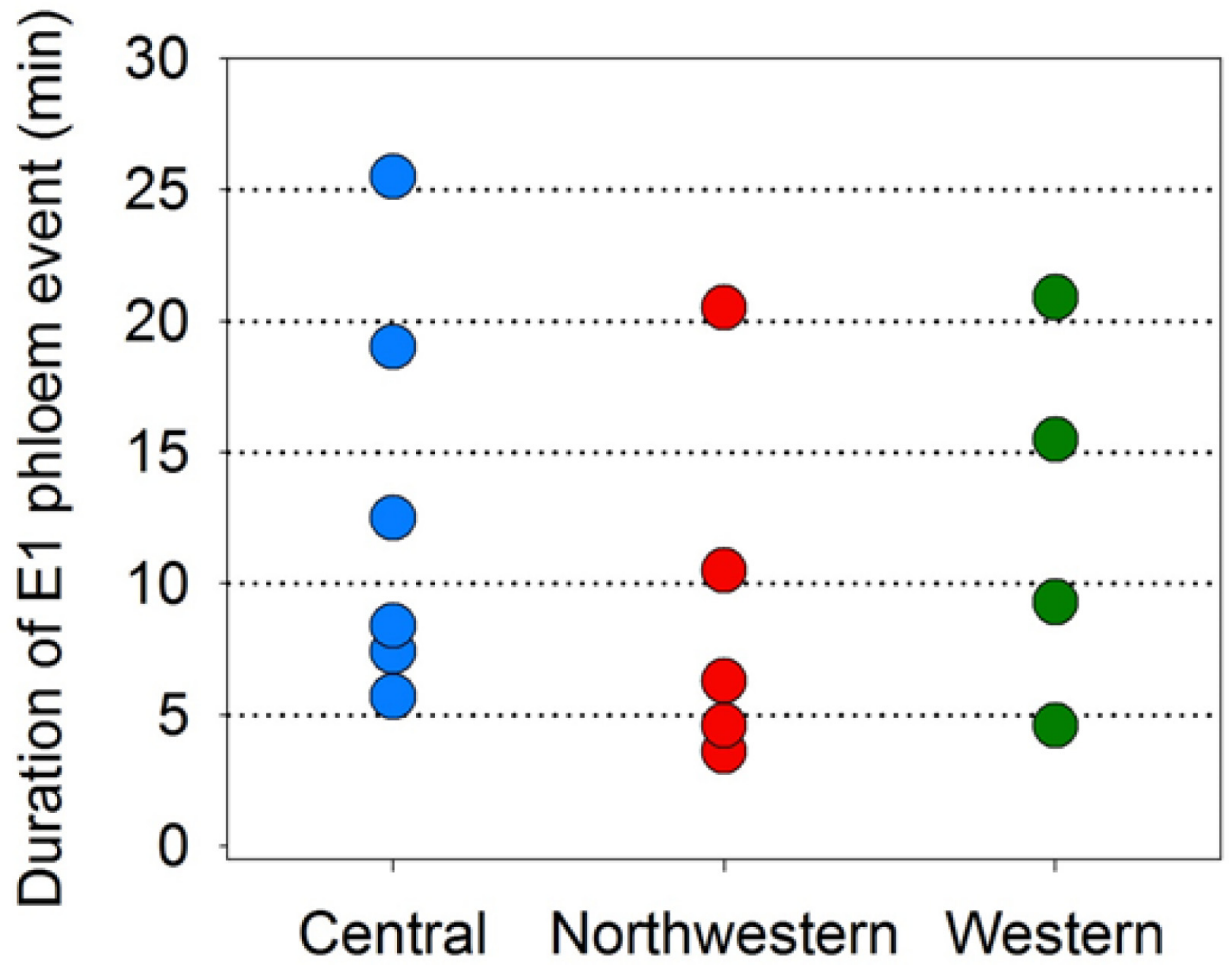
Minimum durations of E1 leading to successful transmission of Lso. Data are shown for those psyllids which (a) exhibited a single E1 event over the duration of an assay (limited additionally to those psyllids for which this single event was shorter than 30 min); and (b) successfully transmitted Lso to potato. A total of 15 psyllids exhibited this specific combination of traits.

Lastly, we examined EPG results from psyllids which exhibited a single event of phloem salivation (E1) to estimate what minimum duration of E1 can lead to Lso-transmission. We limited these data to psyllids that exhibited a single E1 event over the course of an assay, and that exhibited an E1 duration shorter than 30 min (Fig 11). Eight psyllids exhibiting E1 durations less than 10 min produced disease in potato plants, including 3 psyllids having as their sole E1 event and E1 duration of less than 5 min (Fig 11).

## Discussion

The present study is the first to compare feeding behavior and Lso transmission among different haplotypes of potato psyllid. Results showed that the shape and form of the EPG waveforms (C, D, E1, E2, and G) produced by Lso-infected psyllids of the three haplotypes were similar in appearance to waveforms described by Pearson et al. [27] (Figs 2–4), for uninfected psyllids originating from Texas and presumed to be of the Central haplotype [9]. Waveforms were also similar to those reported by Butler et al. [32] in California and Sandanayaka et al. [26] in New Zealand, who presumably used potato psyllids of the Western haplotype [43, 9]. Statistical analysis indicated that there were no significant differences in the number and duration of the different stylet probing behavior events among haplotypes, with the exception of time spent in phloem salivation (E1), which was of longer duration in the Western haplotype than the other two haplotypes (Figs 6B and 10). The similarity of waveforms produced by infected psyllids in this study to the waveforms produced by uninfected psyllids in Pearson et al. [27] suggests that infection with Lso has no major effects on probing behavior. Sandanayaka et al. [26] directly compared waveforms produced by infected and uninfected potato psyllids on tomato, and the only difference found between the two categories of insects was in the intercellular stylet penetration behavior, which was shorter for Lso-infected psyllids than Lso-free insects.

We manipulated the amount of time psyllids were allowed access to the host substrate (IAP) to determine the effects of this variable on probability of Lso-transmission, and in duration and frequency of phloem feeding events. Transmission rates were not statistically different among haplotypes across a range of IAP levels between 1 h and 24 h (Fig 7B). Rates of transmission did vary significantly with IAP (Fig 7B), and exhibited a statistically significant increase between an IAP of 2 h (transmission probability < 10%) and an IAP of 3 h (45 to 60%). Buchman et al. [8] showed that a single potato psyllid was able to induce zebra chip in 47% of plants at an IAP of 6 h, which is a transmission rate somewhat higher than the percentage observed in the present study at a similar IAP (28–44%; Fig 7B). The jump that we observed in successful inoculation between 2 and 3 h IAP (Fig 7B) might be explained by our observation that a psyllid requires on average 3–4 h following the first intercellular stylet probe to reach and salivate into the phloem sieve elements (Fig 5).

We also examined the relationship between IAP and phloem feeding. Particular attention was paid to the time the psyllids spent salivating into the phloem tissue. The phloem salivation (E1) and ingestion (E2) phases (Fig 4) are considered as the probing events during which inoculation and acquisition of pathogens, including Lso, take place [27, 26]. Lso is presumably inoculated into healthy plants during the phloem salivation phase (E1), whereas the bacterium is acquired from infected plants during the phloem ingestion (E2). Probability that a psyllid salivated into phloem tissues (E1) was not affected by haplotype, but did depend upon IAP (Fig 7A); similar to what was observed for transmission probabilities. Probability showed a statistically significant jump between the 2 and 3 h IAP levels (Fig 7A), again similar to results for transmission. Unsurprisingly, the number of separate E1 events per psyllid was larger with increasing IAP, with some psyllids at the longest IAP (24 h) initiating new salivation events over 5 times during a 24 h assay period (Fig 8). Duration of those events, whether total across events for each psyllid (Fig 9A) or expressed as the longest E1 event of the multiple events (Fig 9B) also increased with increasing IAP.

One unexpected result from our IAP assays was the observation that probability of salivation into phloem tissues during an assay was higher than the probability that the assayed psyllid had also transmitted the Lso pathogen, irrespective of IAP (compare Fig 7A and 7B). That is, not all infected psyllids which salivated into phloem also transmitted the pathogen. To examine this observation in more detail, we modeled probability of transmission as a function of how long psyllids salivated into phloem tissues (Fig 10). Probability of transmission increased from a predicted ~0.4 at very short (< 10 min) intervals of phloem salivation, to over 0.95 at the highest duration E1 events. There was no indication that the haplotypes differed statistically in the relationship between E1 and probability of transmission. Somewhat surprising in these results was the high frequency of infected psyllids that failed to transmit Lso despite extremely long intervals of time spent salivating into the phloem (Fig 10: “NO” psyllids). This is consistent with our previous reports that many infected psyllids fail to transmit Lso to new host plants, likely due to the absence of the bacterium in the insect’s salivary glands [44, 35, 45]. Since Lso is transmitted in a propagative, circulative, and persistent manner [35], this pathogen has to be present in the vector’s salivary glands at adequate titer to successfully be transmitted to the host plants. Further research is required to determine what factors influence Lso titer and localization in psyllids, and what factors determine whether infected psyllids are actually infective.

Results of this study further demonstrate the value of electrical penetration graph technology for understanding feeding behaviors of Hemiptera and how those behaviors lead to transmission of plant pathogens [28, 30–31, 26]. Buchman et al. [8] showed that an inoculation access period as short as 6 hr led to development of zebra chip disease in a high percentage of potato plants. That study did not examine actual feeding behavior of psyllids, thus it was not possible to estimate duration of probing activities that actually led to transmission of Lso. The present study, and that of Sandanayaka et al. [26], indicates that even very short intervals of time spent salivating into phloem can lead to transmission of Lso. These results clearly underscore the substantial challenges in managing zebra chip through the control of its insect vector. Potato psyllid can acquire the Lso pathogen from infected tomato plants within 7 min after the onset of ingestion from phloem tissues [26]. The short acquisition time combined with rapid inoculation times could lead to rapid spread of the Lso pathogen within a potato field, especially if insecticides currently used for potato psyllid in potato fields fail to kill the insect rapidly. Products that quickly interfere with probing activities of potato psyllid in addition to having acute toxicity may be of most use for preventing spread of zebra chip disease in potato fields (see Butler et al. [32]).

Lastly, this study provides some insight into the potential importance of the different haplotypes as vectors of Lso within the PNW growing region. While zebra chip was first reported in Mexico in 1994 and in Texas in 2000 [3], the disease did not reach Idaho, Washington, and Oregon until 2011 [5–7]. Psyllids of Central, Western, and Northwestern haplotypes were present in the PNW growing region when the zebra chip outbreak occurred in 2011. Although the Northwestern haplotype predominates in the Columbia Basin of Washington and Oregon [46, 10], presence of Lso in psyllids of this haplotype appears to be rare [10]. This observation has led to speculation that the 2011 zebra chip outbreak in the PNW was due to psyllids other than those of the Northwestern haplotype [3–4], prompting questions as to whether psyllids of this haplotype are efficient vectors of Lso. Results of the present study indicate that psyllids of the Northwestern haplotype were as effective in transmitting Lso to potato as Central and Western psyllids, as shown by the IAP results (Fig 7B) and by non-significant haplotype effects in the logistic analyses (Fig 10). It remains to be determined whether the three haplotypes are also similar in how readily they acquire Lso from infected potato.

## Supporting Information

S1 Table Incidence of plant infection as a function of duration of E1 events (min) for potato psyllid of three haplotypes. Total duration: durations summed across multiple E1 events for each psyllid; longest duration: longest E1 event for each psyllid.
(XLSX)
